# CAREC: Continual Wireless Action Recognition with Expansion–Compression Coordination

**DOI:** 10.3390/s25154706

**Published:** 2025-07-30

**Authors:** Tingting Zhang, Qunhang Fu, Han Ding, Ge Wang, Fei Wang

**Affiliations:** 1 School of Software Engineering, Xi’an Jiaotong University, Xi’an 710049, China; zhang_tt@stu.xjtu.edu.cn (T.Z.); 3122358189@stu.xjtu.edu.cn (Q.F.); 2 State Key Laboratory of Integrated Services Networks, Xidian University, Xi’an 710071, China; 3 School of Computer Science and Technology, Xi’an Jiaotong University, Xi’an 710049, China; dinghan@xjtu.edu.cn (H.D.); gewang@xjtu.edu.cn (G.W.)

**Keywords:** wireless sensing, human action recognition, incremental learning, continual learning

## Abstract

In real-world applications, user demands for new functionalities and activities constantly evolve, requiring action recognition systems to incrementally incorporate new action classes without retraining from scratch. This class-incremental learning (CIL) paradigm is essential for enabling adaptive and scalable systems that can grow over time. However, Wi-Fi-based indoor action recognition under incremental learning faces two major challenges: catastrophic forgetting of previously learned knowledge and uncontrolled model expansion as new classes are added. To address these issues, we propose CAREC, a class-incremental framework that balances dynamic model expansion with efficient compression. CAREC adopts a multi-branch architecture to incorporate new classes without compromising previously learned features and leverages balanced knowledge distillation to compress the model by 80% while preserving performance. A data replay strategy retains representative samples of old classes, and a super-feature extractor enhances inter-class discrimination. Evaluated on the large-scale XRF55 dataset, CAREC reduces performance degradation by 51.82% over four incremental stages and achieves 67.84% accuracy with only 21.08 M parameters, 20% parameters compared to conventional approaches.

## 1. Introduction

Wi-Fi technology has advanced significantly, evolving from early standards to Wi-Fi 6 and the upcoming Wi-Fi 7, with enhanced data rates, coverage, and energy efficiency enabled by technologies like OFDMA and MU-MIMO. Beyond its traditional role in communication, Wi-Fi signals have emerged as a powerful tool for human activity recognition (HAR) [1], leveraging channel state information (CSI) to capture subtle environmental changes caused by human movements, such as the phase shifts induced by limb motions [2] or the amplitude variations from body pose changes [3]. This contact-free paradigm offers unique advantages in cost, privacy, and seamless integration with existing wireless infrastructures, as it eliminates the need for dedicated sensors or user cooperation, positioning Wi-Fi-based HAR as a key enabler for smart homes, healthcare monitoring, and security systems [2,4,5]. For instance, in smart elderly care, Wi-Fi systems can passively monitor daily activities, such as falls, without intrusive cameras, providing timely alerts to caregivers [4].

While Wi-Fi sensing has enabled a wide range of human activity recognition (HAR) tasks, such as smoking detection [6], fall detection [4], gesture recognition [2], tracking [7], and keystroke recognition [8], most existing systems are tailored to specific activities. This task-specific nature limits their flexibility in meeting evolving user demands. For example, a user may initially deploy a smoking detection system at home to support smoking cessation or fire prevention. However, when elderly family members visit or move in, the user might wish to additionally monitor fall incidents to ensure timely caregiver intervention. To accommodate this new need, current approaches typically require training and deploying a separate model specifically for fall detection. As user requirements increase, so does the number of models needed, leading to significant overhead in training, inference, and deployment. This scalability issue motivates the need for a more general and efficient solution—one that can support new activity types without continually expanding the number of models.

**Figure 1:**
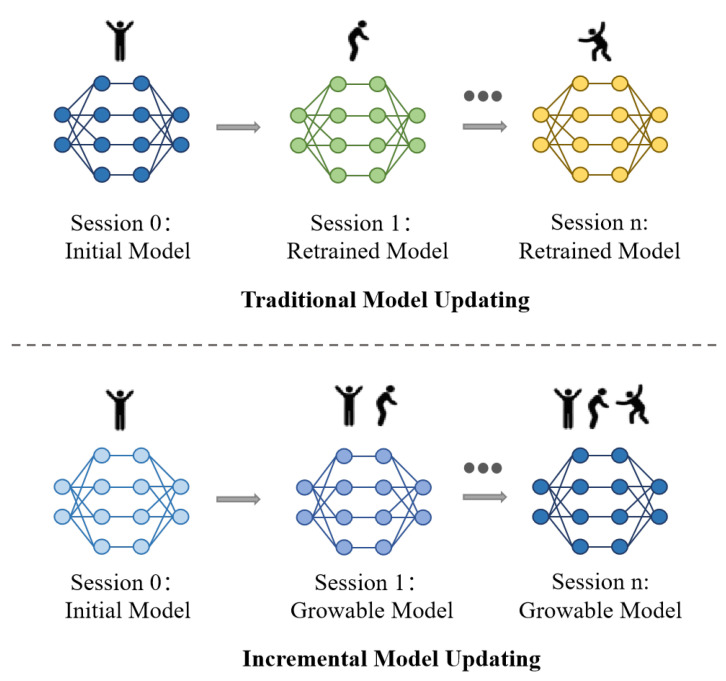
Class-incremental learning for Wi-Fi-based human action recognition model updating. In the traditional scheme, the model is updated in each session using only the current session’s data, which leads to catastrophic forgetting of previously learned action classes. In contrast, class-incremental model updating aims to maintain the ability to recognize actions learned in earlier sessions while learning new action classes from the current session.

Alternatively, one may consider updating the existing model by training it with data from new activity classes, as shown in the top of Figure 1, without increasing the number of deployed models. While this approach reduces inference and deployment costs, it suffers from severe catastrophic forgetting [9]. That is, the model gradually loses the ability to recognize previously learned activities, retaining only the most recently trained ones. For example, as reported in CCS [10], after five rounds of continual model updates, the recognition accuracy on previously seen classes dropped drastically—from an initial 95.07% to just 18.33% in a 55-class classification task, a decline of over 76 percentage points. In such cases, the system becomes practically unusable, as it can no longer reliably recognize earlier activities.

Several recent exploratory efforts, such as WiCAR [11], WECAR [12], and CCS [10], have adopted class-incremental learning strategies to enable model updates for Wi-Fi-based human activity recognition without suffering from catastrophic forgetting, as shown in the bottom of Figure 1. WiCAR introduces a class-incremental learning framework that takes antenna-array-fused image data as input and employs a customized backbone, Wi-RA, enhanced with parallel stacked activation functions. To mitigate forgetting, it combines replay-based training with knowledge distillation and weight alignment, thereby maintaining high recognition performance even after multiple incremental updates. WECAR further extends this idea into a practical end–edge collaborative architecture. By offloading model training and optimization to edge devices (e.g., Jetson Nano) and reserving inference for lightweight end devices (e.g., ESP32), WECAR ensures both continual learning and computational efficiency. It introduces task-specific dynamic model expansion, stability-aware retraining, and a dual-phase hierarchical distillation strategy, achieving strong accuracy while reducing parameter overhead. CCS, on the other hand, envisions a scalable user-facing sensing paradigm in which users can incrementally add new recognition capabilities (e.g., fall detection for elderly care) without uploading data to cloud servers. CCS addresses catastrophic forgetting through local knowledge distillation and weight alignment modules, and demonstrates its effectiveness across multiple wireless modalities, i.e., Wi-Fi, mmWave radar, and RFID. These works collectively highlight the promise of continual learning in wireless sensing; however, these methods either require growing model parameters or face performance degradation over time.

To address the challenges of maintaining performance and parameter efficiency in class-incremental learning, we propose CAREC. CAREC is designed to achieve high adaptability and accuracy in evolving environments by combining the strengths of model expansion and model compression. Specifically, when new activity categories are introduced, CAREC dynamically expands the network by adding a new backbone feature extractor initialized from the previous one. This design allows the model to quickly adapt to new categories while preserving knowledge of previously learned actions. A super-feature extractor aggregates representations from both old and new backbones, enabling rich and discriminative feature learning across tasks. However, continual expansion may lead to model bloat and inefficiency over time. To address this, CAREC introduces a compression phase that reduces the model’s complexity through balanced knowledge distillation. In this phase, a lightweight student model with a single backbone is trained to mimic the behavior of the expanded teacher network. To alleviate class imbalance caused by limited replayed samples, CAREC incorporates a reweighting scheme based on the effective number of samples, ensuring fair and balanced knowledge transfer across all classes. This adaptive expansion–compression mechanism ensures CAREC remains scalable, parameter-efficient, and high-performance in long-term deployment scenarios of Wi-Fi sensing. Our main contributions are summarized as follows:

(1) We introduce CAREC, a class-incremental learning framework tailored for Wi-Fi-based indoor action recognition. CAREC employs a multi-branch architecture that dynamically expands the model by adding new feature extractors while freezing existing ones, effectively preserving previously learned knowledge. To maintain model compactness, a balanced knowledge distillation strategy compresses the network by over 80% (from 105.24 M to 21.08 M parameters).
(2) CAREC combats catastrophic forgetting through modular network design and auxiliary classification heads, prevents uncontrolled model growth via knowledge distillation, and mitigates class imbalance during training with a sample-weighted distillation loss.
(3) Extensive experiments on the large-scale XRF55 dataset demonstrate that CAREC consistently outperforms both classic and state-of-the-art class-incremental learning methods, including iCaRL [11], BiC [13], UCIR [14], BEEF [15], and CCS [10]. CAREC achieves 67.84% accuracy over four incremental stages, highlighting its superior efficiency and suitability for deployment on resource-constrained devices.

The remainder of this paper is organized as follows. Section 2 reviews the related work, covering both WiFi-based action recognition and the state of incremental learning techniques in wireless sensing. Section 3 presents the proposed CAREC method. Section 4 reports the evaluation experiments. Section 5 and Section 6 provide a discussion and the conclusion, respectively.

## 2. Related Work

### 2.1. Wi-Fi-Based Human Activity Recognition

Wi-Fi-based human activity recognition (HAR) utilizes wireless signals to detect and classify human movements by analyzing subtle variations in signal propagation caused by environmental interactions. Early approaches primarily relied on received signal strength indicator (RSSI) for localization tasks, as demonstrated by systems such as RADAR [16] and HORUS [17]. The emergence of channel state information (CSI) techniques marked a significant advancement, enabling fine-grained signal analysis via amplitude and phase measurements across subcarriers. For example, E-eyes [18] introduced CSI histograms as activity fingerprints, achieving low-cost recognition of daily activities, CARM [2] correlated signal dynamics with specific motions using time–frequency analysis, attaining over 96% accuracy in controlled settings. Recent research has embraced deep learning to automate feature extraction from raw CSI data. Approaches based on recurrent neural networks [19], convolutional neural networks [3,20], Transformers [21], and Mamba architectures [22] have shown superior performance without the need for manual preprocessing. Nevertheless, these models cannot typically incrementally learn new activities in real-world deployments.

### 2.2. Incremental Learning in Wireless Sensing

Incremental learning seeks to overcome catastrophic forgetting, the phenomenon where models lose previously learned knowledge when trained on new tasks, through three primary strategies. Regularization-based methods, such as Elastic Weight Consolidation [9] and Memory-Aware Synapses [23], constrain parameter updates on important weights from previous tasks to preserve learned information. Replay-based approaches, including iCaRL [24], maintain a buffer of exemplar samples from old classes and incorporate them during training on new tasks to mitigate forgetting.

Building on these principles, several recent studies have extended class-incremental learning to Wi-Fi-based human activity recognition. WiCAR [11] introduces a replay-based strategy coupled with knowledge distillation to address forgetting and enhances model adaptability through a Wi-RA backbone with parallel stacked activation functions. Additionally, weight alignment is applied to balance the influence of old and new classes during updates. WECAR [12] advances this direction by proposing an end–edge collaborative framework, where edge devices perform dynamic model expansion via task-specific trainable prefixes and selective retraining based on neuron stability. A dual-phase knowledge distillation mechanism ensures the compressed model remains accurate and efficient for deployment on resource-constrained devices such as ESP32. In contrast, CCS [10] emphasizes privacy-preserving model updates, allowing users to locally adapt their models to new sensing needs without transmitting raw data. CCS integrates Herding-based exemplar replay, knowledge distillation, and weight alignment to effectively retain prior knowledge while learning new action classes. However, these methods either require growing model parameters or face performance degradation over time.

### 2.3. Wireless Activity Recognition

Several prior works have investigated the generalization of Wi-Fi-based activity recognition across diverse environments and individuals. Jiang et al. [25] proposed domain-invariant feature learning for environment-independent recognition. Shi et al. [26] introduced CSI enhancement and one-shot learning to improve robustness. Meneghello et al. [27] presented SHARP, a system that achieves person- and environment-independent recognition using commodity access points. These methods focus on generalizability under domain shift, which complements our continual learning approach.

## 3. CAREC

### 3.1. Task Definition

We define the class-incremental learning (CIL) task using the following notation. To simulate real-world streaming data, the training process is divided into *N* incremental stages, with the dataset partitioned as $D_{0},D_{1},\ldots ,D_{N}$. Each stage $D_{i}={\left\{\left(x_{j}^{i},y_{j}^{i}\right)\right\}}_{j=1}^{k_{b}}$ contains $k_{b}$ training samples, where $x_{j}^{i}$ is a data instance belonging to class $y_{j}^{i}\in Y_{i}$, and $Y_{i}$ denotes the label space of the *i*-th stage. For any two stages $i\neq i^{\prime }$, the corresponding label spaces are disjoint, i.e., $Y_{i}\cap Y_{i^{\prime }}=\emptyset$.

The training begins with an initial model $M_{0}$ trained on $D_{0}$. At each subsequent incremental stage *i*, the model $M_{i-1}$ is updated using data from $D_{i}$ to obtain the new model $M_{i}$. The goal of this process is to ensure that, after all incremental stages, the final model can accurately recognize all previously seen action classes. This setting reflects realistic scenarios where recognition systems must adapt to newly emerging user requirements for identifying unseen actions over time.

### 3.2. Methods

CAREC  decouples the continual learning process into two alternating stages: (1) a dynamic expansion phase, which allows the model to integrate novel action classes while preserving previously acquired knowledge, and (2) a lightweight compression phase, which consolidates the learned knowledge into a compact model to ensure deployment efficiency.

**Figure 2:**
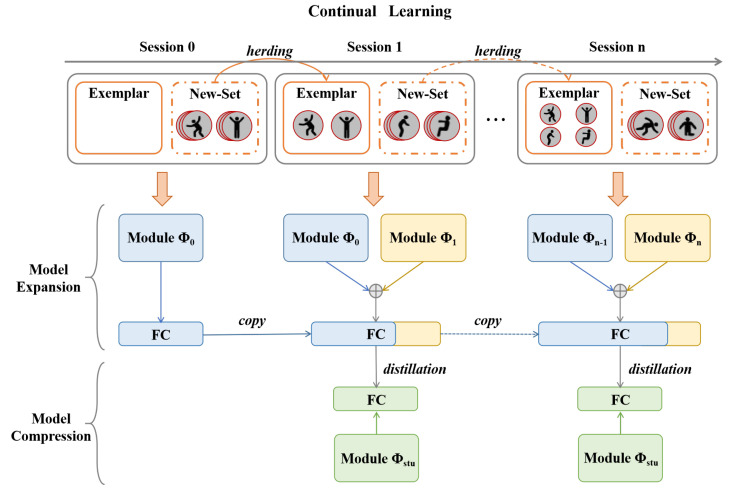
CAREC includes two alternating stages: the model expansion stage and the model compression stage. In the expansion stage, a new backbone and a new classification head are trained to incorporate new classes, while in the compression phase, knowledge from multiple backbones is distilled into a compact student model. The Herding strategy [24] is to select representative exemplars from the latest session. The process supports continual learning without growing the model size.

As illustrated in Figure 2, during each incremental stage, we preserve samples from previously seen classes using data replay, specifically employing the Herding strategy [24] to select representative exemplars (described later in Section 3.2.3). In the expansion phase, the model adopts a multi-backbone architecture to form a modular network, where a super-feature extractor aggregates outputs from multiple feature extractors, each tailored to a specific task, enabling the model to adapt flexibly to newly added categories.

In the compression phase, we apply a balanced knowledge distillation mechanism, in which the expanded network serves as the teacher and a single-backbone student network is trained to replicate its behavior. This step effectively reduces model complexity by eliminating redundant parameters and feature dimensions, while preserving recognition performance, thus maintaining scalability and efficiency for long-term continual learning.

#### 3.2.1. Dynamic Expansion with Super-Feature Extractor

**Figure 3:**
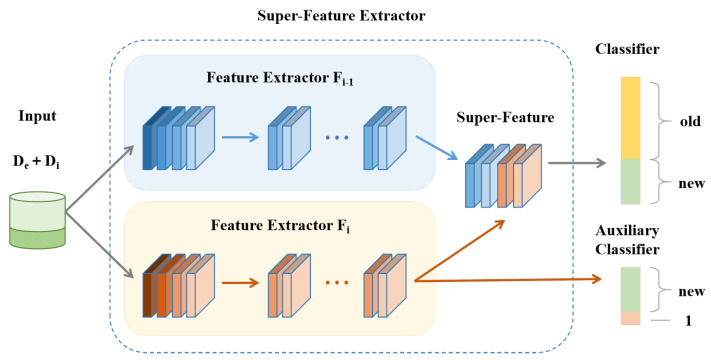
Dynamic expansion with super-feature extractor. In each new session, a dedicated feature extractor is added to learn features for the new classes, which are combined with features from previous sessions and fed into the classifier. The classifier is expanded with new parameters for the newly introduced classes while preserving the parameters for the old classes. Additionally, an auxiliary classifier is introduced, which simplifies the representation of previously seen classes by merging them into a single class.

The model expansion module, as depicted in Figure 3, is designed to enhance adaptability and scalability, particularly under conditions of increasing category diversity and limited data availability. To mitigate catastrophic forgetting, the feature extractor $F_{t-1}$ from the previous stage is frozen at each incremental learning step *t*, preserving learned representations. A new feature extractor $F_{t}$ is then introduced and initialized with the weights of $F_{t-1}$, allowing the model to reuse historical knowledge while adapting efficiently to newly introduced classes.

During training, the newly introduced feature extractor $F_{t}$ and the classifier $C_{t}$ are jointly optimized using the combined dataset $D_{e}\cup D_{i}$, where $D_{e}$ contains exemplar samples from previously learned classes and $D_{i}$ comprises data from newly introduced classes. For a given input sample $x_{i}$, the super-feature $\Phi (x_{i})$ is constructed by concatenating the outputs of both the frozen extractor $F_{t-1}$ and the learnable extractor $F_{t}$:

(1) $$\Phi \left(x_{i}\right)=\left[F_{t-1}\left(x_{i}\right),F_{t}\left(x_{i}\right)\right]$$

This super-feature $\Phi (x_{i})$ is then passed to the classifier $C_{t}$, which produces the final class prediction via the softmax function:

(2) $$p_{C_{t}}\left(x_{i}\right)=\mathrm{Softmax}\left(C_{t}\left(\Phi \left(x_{i}\right)\right)\right)$$

To enhance the model’s ability to distinguish between newly introduced and previously learned action classes, we incorporate an auxiliary classifier $C_{t}^{\mathrm{aux}}$ alongside the primary classifier $C_{t}$. The auxiliary classifier simplifies the representation of old classes by treating all previously seen classes as a single merged class. This design reduces complexity in handling old knowledge while encouraging the model to focus on learning fine-grained distinctions among new classes.

The auxiliary classifier is trained to promote diverse and discriminative feature learning, thereby enhancing the model’s ability in class-incremental settings. The overall loss function combines the objectives of the main and auxiliary classifiers:

(3) $$L=L_{\mathrm{clf}}+\lambda_{a}L_{\mathrm{aux}}$$

where $L_{\mathrm{clf}}$ denotes the loss from the main classifier $C_{t}$, $L_{\mathrm{aux}}$ is the auxiliary classification loss from $C_{t}^{\mathrm{aux}}$, and $\lambda_{a}$ is a weighting coefficient that controls the balance between the two loss components.

This design enables the model to simultaneously leverage stable representations from earlier stages and adaptively learn new representations, improving recognition performance across both old and new action classes.

#### 3.2.2. Lightweight Compression via Balanced Knowledge Distillation

While the model expansion phase effectively preserves performance in class-incremental learning, it inevitably increases the number of model parameters as incremental stages accumulate. To address this scalability issue, we introduce a lightweight compression phase to control model complexity without sacrificing recognition accuracy.

**Figure 4:**
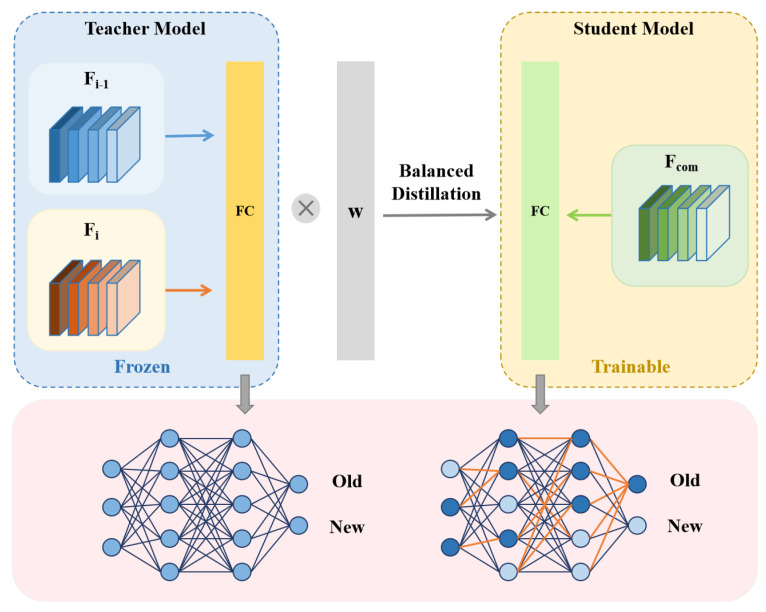
Lightweight compression via balanced knowledge distillation. The super-feature extractor with two backbones serves as the frozen teacher, and a newly initialized single-backbone $F_{\mathrm{com}}\left(x\right)$ acts as the student. Their outputs are aligned via a balanced knowledge distillation loss, where the class-wise weights *w* are inversely proportional to effective sample counts and reduce bias toward majority classes and enhance minority class learning. This enables the compressed student to maintain strong performance with reduced complexity.

As shown in Figure 4, we employ a newly initialized single-backbone network $F_{\mathrm{com}}$ as the student model, while the super-feature extractor Φ, composed of two backbone networks, serves as the teacher model. During training, the parameters of Φ are fully frozen, and knowledge distillation is applied as a simple yet effective means of achieving model compression.

In the context of class-incremental learning, the exemplar set $D_{e}$ contains only a small number of samples from previously learned classes, while the current stage dataset $D_{i}$ is relatively large. This imbalance naturally leads to a long-tailed distribution across classes. However, traditional knowledge distillation frameworks, although effective in general settings, tend to exhibit bias toward head classes on imbalanced datasets [28]. In such cases, the predictive knowledge from tail classes is overwhelmed, causing the student model to perform suboptimally under the influence of a biased teacher model.

To address the classification bias caused by the imbalance between new and old class sample counts in $\left\{D_{e},D_{t}\right\}$, we introduce a reweighting scheme based on the effective number of samples. This scheme adjusts the distillation process to emphasize under-represented classes, thereby producing a class-balanced loss. Specifically, we compute the effective number of samples $E_{n}$ for each class as follows:

(4) $$E_{n}=\frac{1-\beta^{n}}{1-\beta }$$

where *n* is the number of training samples for a given class, and β∈(0,1) is a hyperparameter that controls the smoothness of the weighting curve. Based on this, we construct a weight vector $\omega :=1/E_{n}$, where each class *k* is assigned a weight inversely proportional to its effective sample size.

The resulting balanced knowledge distillation (BKD) loss is defined as follows:

(5) $$L_{\mathrm{BKD}}=\mathrm{KL}\left(\omega ⊗\mathrm{Softmax}\left(\Phi_{t}\left(x\right)\right)\|\mathrm{Softmax}\left(F_{\mathrm{com}}\left(x\right)\right)\right)$$

where $\Phi_{t}\left(x\right)$ denotes the teacher’s output, and $F_{\mathrm{com}}\left(x\right)$ denotes the student’s output. The element-wise product ω⊗Softmax(·) reweights the soft targets class-wise. The weight ω is monotonically decreasing with the number of samples *n*, meaning that classes with fewer samples are assigned larger weights. This weighting mechanism ensures that minority classes receive larger gradients during training, thus mitigating bias toward dominant classes.

By applying this balanced distillation strategy, the compressed model maintains only a single backbone, making it comparable in parameter count to conventional single-backbone architectures. This not only ensures efficient storage and computation but also prevents performance degradation on new classes due to interference from redundant components retained in previous stages.

#### 3.2.3. Data Replay

Data replay is an implementation detail of our proposed CAREC, used to select representative data from the previous stage to mitigate the model’s forgetting of old action classes. CAREC adopts the Herding strategy [24] as the data replay method. For an action class with samples $X=\left\{x_{1},x_{2},\ldots ,x_{n}\right\}\subset D_{i-1}$ from the previous stage, the mean feature vector μ of the class is calculated as follows:

(6) $$\mu =\frac{1}{n}\sum_{x\in X}\Phi \left(x\right)$$

Considering memory constraints in real-world terminal systems, we retain only *m* exemplars per class. To select the *m* samples closest to the class mean μ, we apply Equations (7) and (8) to recursively select the top *m* representative samples as exemplars.

(7) $$\begin{array}{cc} d_{1} & =\underset{x\in X}{\arg\min}∥\mu -\Phi (x)∥ \end{array}$$

(8) $$\begin{array}{cc} d_{m}|\left(m>1\right) & =\underset{x\in X}{\arg\min}∥\mu -\frac{1}{k}\left[\Phi \left(x\right)+\sum_{i=1}^{m-1}\Phi \left(d_{i}\right)\right]∥ \end{array}$$

For each class in the previous-stage training set $D_{i-1}$, we select *m* such samples, denoted as $D_{e}$. The exemplar set $D_{e}$ is then combined with the new training data $D_{i}$ to form the training set for the *i*-th incremental stage.

## 4. Evaluation

### 4.1. Dataset

To validate the effectiveness of our proposed CAREC, we adopt the XRF55 dataset [5]. As shown in Table 1, XRF55 stands out in terms of the diversity of action classes, categories, and action samples, thereby better meeting the demands of real-world incremental learning scenarios where new action classes continuously emerge. Specifically, XRF55 comprises 55 action categories spanning five major types of indoor human activities: human–object interaction, human–human interaction, fitness activities, body movements, and human–computer interaction. These action classes encompass not only common daily actions, such as walking and sitting, but also more complex interactive behaviors, like hula hooping and playing musical instruments. Another significant advantage of the XRF55 dataset is its participant diversity. Each action category is performed by 39 volunteers with varying body types, and each volunteer contributes 20 samples per action. This enhances the representativeness of the dataset and enables the model to learn more generalizable features. Following the official data split, we use the first 14 samples of each action from each volunteer as the training set, and the remaining 6 samples as the test set. Note that all evaluations in this study are conducted using data from Scene 1 of the XRF55 dataset, which includes 55 action classes, 30 volunteers, and a total of 33,000 samples. We did not conduct data preprocessing and data augmentation on the XRF55 dataset in our evaluation.

To evaluate CAREC, we follow the experimental protocol in CCS [10] by partitioning the XRF55 dataset into five non-overlapping subsets $\left\{D_{i}|i=0,1,2,3,4\right\}$, each containing distinct action classes. Specifically, these subsets include 15, 10, 10, 10, and 10 action classes, respectively, corresponding to five incremental learning stages. Each stage simulates a real-world scenario where users demand recognition of new action classes. In the initial stage, the model $M_{0}$ is trained using $D_{0}$, which contains 15 action classes. In the subsequent four incremental stages, 10 new action classes are introduced at each stage. To mitigate catastrophic forgetting, we adopt the Herding method [24] as the data replay strategy. Specifically, in the *i*-th incremental stage, 30 representative samples are selected from each action class in the previous dataset $D_{i-1}$ and stored in a replay buffer $D_{e}$. During the training of model $M_{i}$, both the current dataset $D_{i}$ and the replay buffer $D_{e}$ are used jointly to preserve prior knowledge while learning new action class representation.

### 4.2. Evaluation Metrics

We use action recognition accuracy (${\mathrm{Accuracy}}_{i}$) as the evaluation metric to assess the model’s performance at each incremental stage. The calculation is defined as follows:

(9) $${\mathrm{Accuracy}}_{i}=\frac{\sum_{j=1}^{n}I\left(y_{\mathrm{pred}}\left[j\right],y_{\mathrm{gt}}\left[j\right]\right)}{n}$$

where *n* denotes the total number of test samples for all action classes supported by the model at the current stage, i.e., the cumulative size of test sets from $D_{0}$ to $D_{i}$. Here, $y_{\mathrm{pred}}\left[j\right]$ and $y_{\mathrm{gt}}\left[j\right]$ represent the predicted and ground truth labels of the *j*-th sample, respectively. $I(y_{\mathrm{pred}}\left[j\right],y_{\mathrm{gt}}\left[j\right])$ outputs 1 if $y_{\mathrm{pred}}\left[j\right]$ equals to $y_{\mathrm{gt}}\left[j\right])$, otherwise it outputs 0.

Additionally, we use ACCN, a metric proposed in CCS [10], to evaluate overall performance:

(10) $${\mathrm{ACCN}}_{i}={\mathrm{Accuracy}}_{i}\times \sum_{j=0}^{i}N_{j}$$

where $N_{j}$ denotes the number of action classes in dataset $D_{j}$. ${\mathrm{ACCN}}_{i}$ serves as a comprehensive metric that jointly reflects the classification accuracy and the total number of action classes the model can recognize. As described in CCS, ACCN captures the increasing value of the model as new classes are incrementally added, providing a more holistic evaluation of the model’s utility in real-world scenarios.

### 4.3. Experimental Details

All experiments were conducted on a server equipped with four NVIDIA GeForce RTX 3090 (manufactured by NVIDIA Corporation, based in Santa Clara, CA, USA). 24 GB GPUs, Ubuntu 18.04.5 LTS, CUDA 11.7, Python 3.9, and PyTorch 1.13.1. The key hyperparameters and training configurations are listed in Table 2. We employed the Stochastic Gradient Descent (SGD) optimizer with a batch size of 128 to balance computational efficiency and gradient stability. The model was trained for 150 epochs in the expansion phase and 130 epochs in the compression phase. The initial learning rate was set to 0.1 and scheduled using MultiStepLR, which decayed the learning rate by a factor of 0.1 at the 80th and 120th epochs during expansion. This learning rate strategy facilitated effective convergence and mitigated overfitting. Additionally, a weight decay of $2\times 10^{-4}$ was applied to regularize the model and promote parameter stability throughout training. We experimentally adopt temporal UNet [33] as the backbone network. The analysis of the time and space complexity of CAREC based on this backbone is provided in the Appendix B, on pages 19–20.

**Figure 5:**
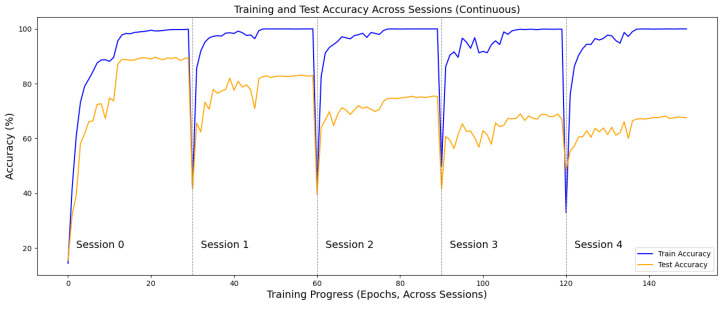
Learning curves of training and test accuracy across sessions. Accuracies increase within each session and drop at the start of the next due to new classes, with test accuracy showing a gradual decline from accumulated forgetting.

As shown in the learning curves (Figure 5), both the training and test accuracies within each session gradually increase and eventually saturate. At the beginning of a new session, the accuracies drop significantly due to the introduction of unseen action classes and then rise again as the model learns the new categories. It can be observed that the training accuracy achieves satisfactory levels in each session, while the test accuracy shows a decreasing trend across sessions. This is because, as more sessions are added, the proportion of previously learned knowledge increases, and more of it is subject to forgetting over time.

### 4.4. Results

We evaluate the proposed CAREC through comparative experiments across multiple incremental training stages against several representative class-incremental learning methods and baseline approaches. Specifically, methods including iCaRL [24], BiC [13], UCIR [14], BEEF [15], and CCS [10] are implemented following their original configurations as described in the respective literature. As a baseline, we take a straightforward approach that updates the model at each stage using only data from new classes without employing any additional incremental learning strategies. The comparative results are summarized in Table 3.

As shown in Table 3, without any incremental learning strategies, the baseline method suffers severe performance degradation, with accuracy dropping from 89.67% in the initial stage to only 16.25% in the final stage, indicating that the model almost completely forgets the ability to recognize actions seen in former stages. In contrast, applying incremental learning techniques significantly improves the model’s recognition capability. For example, the accuracy of iCaRL [24] across the five sessions is 90.41, 74.09, 66.90, 63.68, and 63.49, while CAREC achieves 89.44, 81.36, 75.00, 63.46, and 67.84, respectively. Both methods start from a comparable performance level at Session 0. As the incremental learning progresses, CAREC outperforms iCaRL in Session 1, 2, and 4, while showing comparable performance in Session 3. Notably, our proposed method, CAREC, achieves the best performance with an accuracy of 67.84% on all 55 action classes in the final stage. This demonstrates CAREC’s superior effectiveness in mitigating catastrophic forgetting during model updates. Since UCIR is the best-performing baseline method, we ran five trials each for the baseline, UCIR, and CAREC, and conducted *t*-test analyses. The experiments demonstrate that our method significantly outperforms these approaches. The detailed *t*-test results are provided in the Appendix A on pages 18–19.

**Figure 6:**
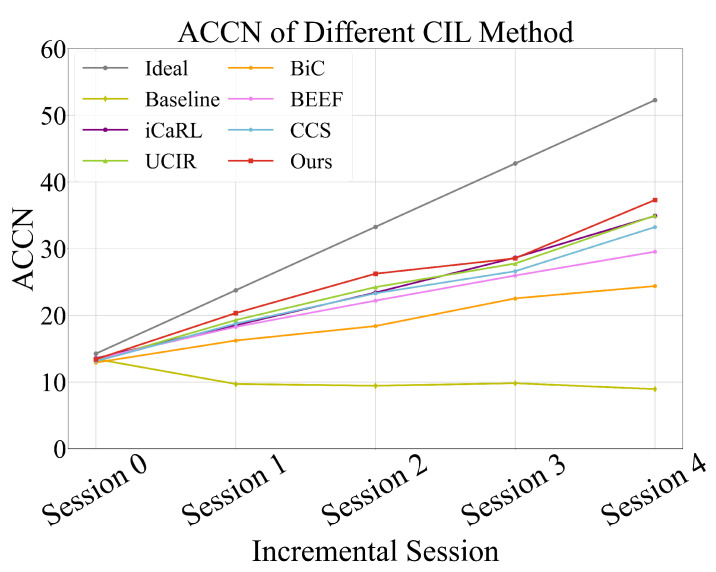
CAREC achieves the best ACCN and demonstrates the advantage in continual stages as more action classes are incrementally added.

Figure 6 illustrates the ACCN metric across incremental stages. In an ideal scenario (denoted as Ideal), the model’s value would exhibit a linear upward trend as new action classes are introduced. However, in real-world applications, the model tends to forget previously learned knowledge, causing fluctuations in ACCN. While the performance differences between methods are relatively small in the initial stage, CAREC demonstrates a significant advantage in subsequent stages as more action classes are incrementally added. This indicates that CAREC effectively adapts to new tasks while maintaining knowledge of previous ones. Its consistent performance highlights strong adaptability and stability in dynamic learning environments, making it better suited for real-world scenarios where action classes continuously increase.

### 4.5. Ablation Study

In this work, we use data replay, model expansion, and balanced knowledge distillation and compression techniques to implement model structure-based class-incremental learning. To verify the effectiveness of the scheme, the following ablation experiments are conducted.

#### 4.5.1. Impact of Data Replay

Data replay plays a pivotal role in our proposed method, aiming to alleviate catastrophic forgetting by reusing data from previous tasks during the learning process. In our evaluated XRF55 dataset, each action class comprises samples from 30 volunteers. To accommodate practical hardware constraints and system deployment requirements, we adopt the Herding strategy [24] to select 30 representative samples per known class after each training stage for replay.

As shown in Table 4, the absence of replay samples leads to a dramatic performance drop. Without data replay, the model’s action recognition accuracy on Wi-Fi data declined sharply from 89.44% in the initial session to 15.96% in the final incremental stage, a decrease of 73.48 percentage points. In contrast, when replay samples were incorporated, the model’s performance degradation was significantly mitigated. After four incremental learning stages, during which 40 new action classes were added, the average recognition accuracy decreased from 89.44% to 67.84%, representing a much smaller decline of only 21.60 percentage points.

**Figure 7:**
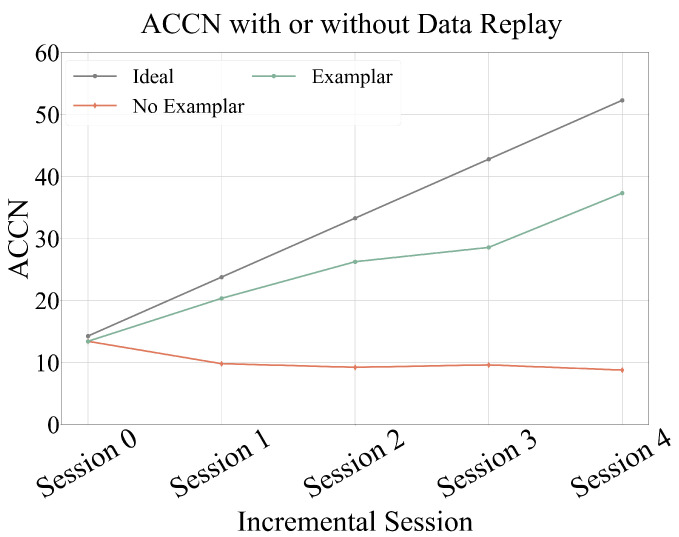
ACCN for data replay ablation study. CAREC with exemplar data replay significantly outperforms the version without replay, demonstrating the effectiveness of data replay in mitigating catastrophic forgetting.

Figure 7 depicts the ACCN values of the model at each training session with and without data replay. Without replay examples, the model’s ACCN values remained at a low level across all stages and even showed a downward trend. Conversely, when replay examples were used, the model’s value at each stage increased significantly. This clearly demonstrates that introducing a small number of typical samples of known categories during incremental training can substantially reduce model performance degradation and enhance the model’s overall value.

#### 4.5.2. Impact of Model Expansion

**Figure 8:**
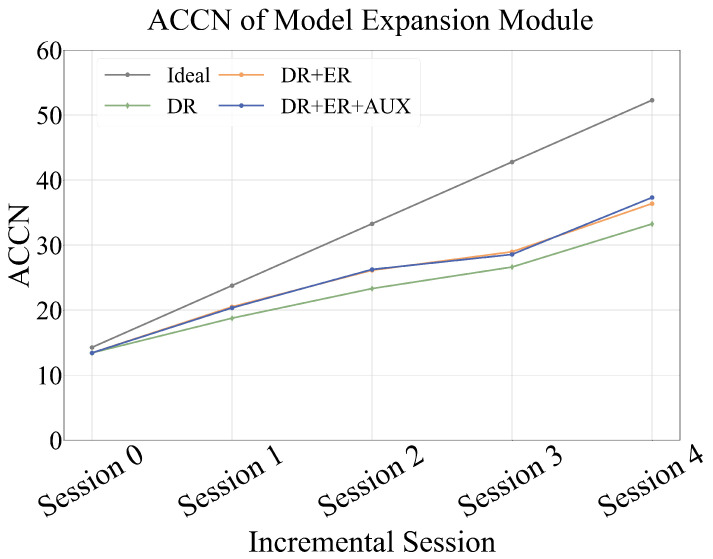
ACCN of the model expansion module. The data replay (DR), when combined with extended representation (ER) and auxiliary losses, achieves the best performance among all configurations.

Under the data replay setting, we conducted an ablation study on the model expansion module to assess the contributions of the extended representation and auxiliary loss strategies. The experimental results are presented in Table 5. When only data replay was applied—without incorporating either extended representation or auxiliary loss—the model achieved an accuracy of 60.45% in the final incremental session. Introducing the extended representation alone led to a noticeable improvement, boosting the Session 4 accuracy to 66.15%, a gain of 5.7 percentage points. When both extended representation and auxiliary loss were employed, the performance improved further, with the final accuracy reaching 67.84%, representing an additional 1.69 percentage point improvement. The ACCN curve in Figure 8 further validates this conclusion.

These results demonstrate that the combination of extended representation and auxiliary loss strategies effectively mitigates performance degradation during class-incremental learning, enhancing the model’s ability to retain and integrate knowledge across incremental sessions.

#### 4.5.3. Selection of Model Compression Methods

To evaluate the impact of different model compression strategies on class-incremental learning performance, we compared conventional knowledge distillation (KD) with balanced knowledge distillation (BKD). The results, summarized in Table 6, show that both methods achieved the same accuracy of 89.44% in the initial session (Session 0), indicating equivalent performance at the starting point. However, as incremental sessions progressed, BKD consistently outperformed conventional KD. By comparing the model’s action recognition accuracy between the initial session and the final incremental session (Session 4), we observed that the KD-based model experienced a performance drop of 25.7 percentage points, whereas the BKD-based model showed a smaller decline of only 21.6 percentage points, resulting in a 4.1-point improvement in mitigating performance degradation.

**Figure 9:**
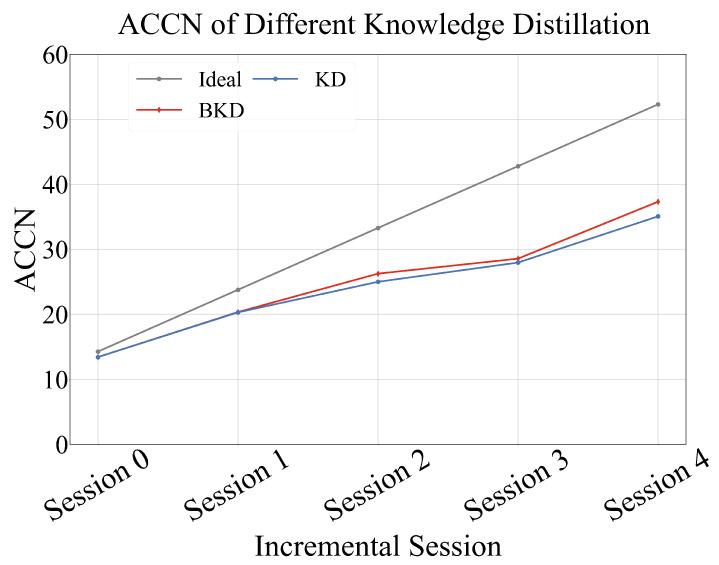
ACCN of different knowledge distillation. The proposed balanced knowledge distillation (BKD) outperforms conventional knowledge distillation (KD), demonstrating its effectiveness.

Figure 9 illustrates the curve of ACCN change when model compression is achieved using the knowledge distillation strategy. The results in the figure show that the value of the model using the balanced knowledge distillation compression method is relatively higher.

This performance gain is primarily attributed to BKD’s ability to address the class imbalance between new and old classes during distillation, thereby preserving knowledge from previously learned classes more effectively throughout the incremental learning process.

#### 4.5.4. Impact of Model Compression

The primary objective of model compression is to limit the growth of model size while retaining maximum performance, enabling deployment in resource-constrained environments and ensuring efficient utilization of computational resources. To validate the effectiveness of the balanced knowledge distillation (BKD)-based compression strategy proposed in this work, we conducted a series of ablation experiments.

As presented in Table 7, without compression, the model consisted of 105.24 M parameters, and its average recognition accuracy for previously learned action categories dropped from 89.44% to 70.09% over the course of five incremental sessions, reflecting a degradation of 18.35 percentage points. In contrast, when the proposed compression module was applied, the model size was significantly reduced to 21.08 M parameters, achieving an 80% reduction (approximately 5× compression). Despite this drastic reduction in size, the recognition performance remained stable, with accuracy reaching 67.84% in the final session, only 2.25 percentage points lower than the uncompressed model.

**Figure 10:**
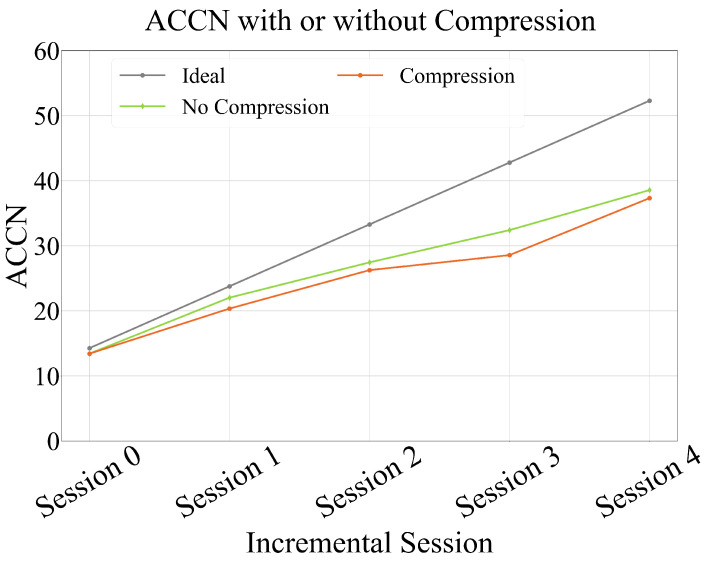
ACCN with or without compression. After compression, the model achieved 67.84% accuracy in the final session—only 2.25% points lower than the uncompressed model—while the model size was reduced to 21.08 M parameters, achieving an 80% reduction.

Figure 10 further illustrates the ACCN curves before and after compression. The two curves are nearly overlapping across all incremental sessions, indicating that the proposed compression strategy effectively preserves overall model utility during class-incremental learning.

The experimental results show that although the model parameter quantity was significantly reduced, the model performance degradation in class-incremental learning was small. This result indicates that the balanced knowledge distillation-based model compression technology can maintain high classification performance while significantly reducing the parameter quantity, fully demonstrating the effectiveness of the proposed compression module. This finding provides important practical evidence for deploying class-incremental learning models in resource-constrained environments and validates the advantages of model compression technology in balancing model efficiency and performance.

## 5. Discussion

Several challenges remain due to the complexity and variability of real-world scenarios, warranting further investigation in future work.

(1) Generalization Across Users and Environments: The incremental learning tasks and evaluations conducted in this paper were performed under specific environmental and user settings, without explicitly addressing generalization across different users or environments. This limitation restricts the applicability of the model in real-world deployments [34]. Domain shifts caused by changes in space layout, antenna configuration, or subject physiology may significantly affect performance. Although our framework supports adaptation over time, it currently assumes a consistent deployment domain. Future research could explore extending continual learning with domain adaptation techniques and environment-invariant representation learning [25,26,27] to handle environments and user shifts.
(2) Bottlenecks and Noisy Environments: CAREC has the generalization ability in the incremental action classes over time, but it has not yet been rigorously evaluated in extreme conditions such as crowded and noisy spaces. CSI signals are susceptible to various sources of distortion, including environmental interference, device placement variability, and multipath fading, which can degrade the discriminative quality of the extracted features. Future work can integrate noise-resilient preprocessing techniques, denoising autoencoders, and adversarial robustness training to enhance the system’s reliability in real-world deployments.
(3) Temporal Feature Extractor: Although CAREC achieves promising results in continual Wi-Fi action recognition by balancing model expansion and compression, it still has several limitations. First, the current feature extraction pipeline simply concatenates outputs from multiple backbones without explicitly modeling temporal dependencies, which may limit its ability to capture long-range temporal patterns in complex actions. Second, the lack of explicit spatial, channel-wise, or multi-scale attention mechanisms may reduce the model’s robustness and discriminative power in noisy or cluttered environments. To address these limitations, in future work we plan to enhance the feature extraction capability of CAREC by incorporating advanced temporal and spatial attention mechanisms. Inspired by the later temporal attention mechanism [35], we will explore emphasizing the contribution of later frames in the CSI sequence to better capture critical information in long-duration actions. Additionally, motivated by the multi-attention optimization network [36], we aim to integrate spatial, channel-wise, and multi-scale attention modules into the backbone and super-feature extractor, improving feature robustness and discrimination under complex wireless conditions. Furthermore, the idea of deep historical long short-term memory [37] can also inspire us to augment the temporal modeling capability of CAREC. Specifically, by incorporating a historical feature update mechanism into the super-feature extractor, CAREC may better retain and utilize long-term temporal dependencies across frames, enhancing the recognition of actions with subtle and cumulative temporal dynamics.
(4) Multi-modal Data Fusion: Our current implementation relies solely on Wi-Fi Channel State Information (CSI) as the data source. Although Wi-Fi sensing offers the advantage of device-free interaction, it can suffer from limitations in complex settings due to signal attenuation, occlusion, and multi-path effects. In future work, we plan to explore multi-modal sensor fusion to enhance the model’s robustness and perceptual capability. For instance, integrating inertial sensing data (e.g., accelerometers and gyroscopes from smartphones or wearables [22]) could improve fine-grained motion recognition. In scenarios where privacy concerns permit, incorporating camera-based visual data can further support complex activity understanding, particularly in cases involving subtle gestures or multi-person interactions, by providing rich contextual information that complements wireless signals.

## 6. Conclusions

In this paper, we proposed CAREC, a class-incremental learning framework designed for Wi-Fi-based indoor action recognition. Extensive experiments on the XRF55 dataset demonstrated its superior performance over existing methods, highlighting the framework’s potential for practical deployment in wireless sensing tasks.

## Figures and Tables

**Table 1:** We adopt the XRF55 dataset for its diversity in both the number and types of action classes, better meeting the demands of real-world incremental learning scenarios where new action categories continuously emerge.

Dataset	Action Class	Volunteer Number	Action Sample	Action Category
Widar3.0 [19]	16	16	17,000	2
WiAR [29]	16	10	4800	1
ARIL [30]	6	1	1394	1
MM-Fi [31]	27	40	9 h	2
UI-HAR [32]	6	6	720	1
XRF55 [5]	55	39	42,900, 59.58 h	5

**Table 2:** Training hyperparameter.

Training Parameter	Value
Batch Size	128
Training Epochs	150
Learning rate	0.1
Compression Epochs	130
Compression Learning Rate	0.1
Optimizer	SGD
Weight Decay	$2\times 10^{-4}$
Learning Rate Milestones	[80, 120]
Learning Rate Decay Factor	0.1
Loss Function	Cross-Entropy Loss

**Table 3:** CAREC achieves the best performance with an accuracy of 67.84% on all 55 action classes in the final stage, demonstrating its superior effectiveness in mitigating catastrophic forgetting during model updates. Hyperpatameters of all methods are provided in the json files in https://github.com/aiotgroup/carec/tree/main/exps (accessed on 6 June 2025).

Method	Session 0	Session 1	Session 2	Session 3	Session 4
Baseline	89.67	38.84	26.97	21.83	16.25
iCaRL [24]	90.41	74.09	66.90	63.68	63.49
BiC [13]	86.11	64.91	52.56	50.10	44.35
UCIR [14]	88.07	77.16	69.24	61.73	63.58
BEEF [15]	89.81	73.11	63.46	57.72	53.73
CCS [10]	87.48	75.02	66.59	59.16	60.45
CAREC (ours)	89.44	81.36	75.00	63.46	67.84

**Table 4:** Results of ablation experiments with data replay.

Data Replay?	Session 0	Session 1	Session 2	Session 3	Session 4
no	89.44	39.22	26.35	21.36	15.96
yes	89.44	81.36	75.00	63.46	67.84

**Table 5:** In CAREC, extended representation and auxiliary loss strategies effectively mitigate performance degradation during class-incremental learning.

Extended Representation	Auxiliary Losses	Session 0	Session 1	Session 2	Session 3	Session 4
×	×	87.48	75.02	66.59	59.16	60.45
✓	×	89.44	82.02	74.63	64.37	66.15
✓	✓	89.44	81.36	75.00	63.46	67.84

**Table 6:** Comparative experiments on model compression methods.

Method	Session 0	Session 1	Session 2	Session 3	Session 4
KD	89.44	81.18	71.41	62.12	63.74
BKD	89.44	81.36	75.00	63.46	67.84

**Table 7:** In CAREC, compression strategy effectively preserves model accuracy and largely reduces model parameters.

Compressed?	# Parameters	Session 0	Session 1	Session 2	Session 3	Session 4
no	105.24 M	89.44	88.07	78.40	72.00	70.09
yes	21.08 M	89.44	81.36	75.00	63.46	67.84

## Data Availability

No new data were created. The code is available at https://github.com/aiotgroup/carec/ (accessed on 6 June 2025).

## Appendix A. *t*-Test Significance

We conducted five independent training and testing runs for CAREC, the second-best performing UCIR, and the baseline, in order to perform the *t*-test significance analysis.

**Table A1:** Accuracy (%) of CAREC and UCIR across five sessions (5 runs each).

Method	Session 0	Session 1	Session 2	Session 3	Session 4
CAREC	89.44	81.36	75.00	63.46	67.84
	88.30	82.13	75.19	64.00	67.17
	88.85	81.76	75.92	66.43	68.46
	89.11	83.29	76.05	61.30	67.36
	89.44	81.67	75.03	61.63	67.65
UCIR	88.07	77.16	69.24	61.73	63.58
	88.67	79.04	70.05	60.14	62.49
	88.93	77.02	69.16	59.84	61.07
	88.41	79.22	69.57	61.41	63.70
	87.81	78.58	69.87	59.48	61.65
Baseline	89.67	38.84	26.97	21.83	16.25
	86.93	38.36	26.57	21.99	16.00
	87.44	38.18	26.08	20.75	16.14
	88.96	38.76	26.67	22.02	16.47
	88.37	38.67	26.65	21.73	16.08

**Table A2:** Welch’s *t*-test results comparing CAREC and UCIR accuracies for each session.

Session	t-Statistic	*p*-Value	Significance
1	5.6104	0.0012	Significant
2	16.3495	<0.0001	Significant
3	1.0323	0.3404	Not Significant
4	5.5218	0.0013	Significant

**Table A3:** Welch’s *t*-test results comparing CAREC and Finetune accuracies for each session.

Session	t-Statistic	*p*-Value	Significance
1	121.4554	<0.0001	Significant
2	181.9547	<0.0001	Significant
3	43.7360	<0.0001	Significant
4	216.8109	<0.0001	Significant

The Welch’s *t*-test statistic is calculated as follows:

(A1) $$t=\frac{{\bar{X}}_{1}-{\bar{X}}_{2}}{\sqrt{\frac{s_{1}^{2}}{n_{1}}+\frac{s_{2}^{2}}{n_{2}}}}$$

where

- ${\bar{X}}_{1}$ and ${\bar{X}}_{2}$ are the sample means of the two groups;
- $s_{1}^{2}$ and $s_{2}^{2}$ are the sample variances;
- $n_{1}$ and $n_{2}$ are the sample sizes.

As shown in Table A2 and Table A3, CAREC significantly outperforms both UCIR and Finetune in sessions 1, 2, and 4 (p<0.05). In session 3, the difference between CAREC and UCIR is not statistically significant (p=0.3404), while CAREC still significantly outperforms Finetune in the same session (p<0.0001).

## Appendix B. t-Test Significance

In this section, we present a detailed analysis of the computational and memory complexity of the proposed 1D U-Net model. We report both theoretical asymptotic complexity (O-notation) and practical metrics (FLOPs and parameter count).

### Appendix B.1. Space Complexity (Parameter Count)

The space complexity of the model is primarily determined by the number of trainable parameters. We compute the parameter count layer by layer.

#### Key Modules and Their Parameter Count

**Table A4:** Parameter count of main modules.

Module	Formula	Parameters
double_conv(in, out)	2×[in×out×3+out(BN)]	2×(3·in·out+2·out)
inconv(52, 128)	double_conv(52, 128)	40,192
down1(128, 256)	double_conv(128, 256)	197,120
down2(256, 512)	double_conv(256, 512)	787,456
down3(512, 1024)	double_conv(512, 1024)	3,146,240
down4(1024, 1024)	double_conv(1024, 1024)	6,293,504
up1(2048, 512)	ConvTranspose1d(1024,1024) + double_conv(2048, 512)	8,407,040
up2(1024, 256)	similar to up1	2,101,248
up3(512, 128)	similar to up1	525,312
up4(256, 128)	similar to up1	131,200
outconv(128, 512)	128×512×1	65,536

The total number of parameters is approximately 21.6 M, with the largest contributions coming from the down4 and up1 modules (about 70% of the total).

**Table A5:** Approximate FLOPs per module.

Module	FLOPs (Millions)
inconv(52→128)	40
down1	98
down2	196
down3	393
down4	390
up1	917
up2	262
up3	66
up4	33
outconv	66
Total	2500 (2.5 GFLOPs)

### Appendix B.2. Time Complexity (FLOPs)

We estimate the time complexity based on forward-pass floating point operations (FLOPs). Assuming an input tensor of shape (B,C,L) with batch size B=1 and sequence length L=1000, we analyze the main operations.

#### Appendix B.2.1. Key Operation Complexity

- Convolution: FLOPs=B×L×in×out×k;
- Transposed convolution: FLOPs=B×L×in×out×k;
- Other operations (pooling, BN, upsampling): negligible (<5%).

#### Appendix B.2.2. Per-Module FLOPs (Approximate, for (*B* = 1, *L* = 1000)

The total FLOPs for a single-input sample is approximately 2.5 GFLOPs, dominated by the up1, down3, and down4 modules.

### Appendix B.3. Complexity Summary

#### Asymptotic Complexity

- Time complexity: $O(L\cdot C^{2}\cdot K)$, where *L* is the sequence length, *C* is the channel width, and *K* is the kernel size.
- Space complexity: $O(C^{2}\cdot K\cdot N_{\mathrm{layers}})$.
